# Growth Factors in the Carotid Body—An Update

**DOI:** 10.3390/ijms21197267

**Published:** 2020-10-01

**Authors:** Elena Stocco, Silvia Barbon, Cinzia Tortorella, Veronica Macchi, Raffaele De Caro, Andrea Porzionato

**Affiliations:** Department of Neurosciences, Institute of Human Anatomy, University of Padova, 35121 Padova, Italy; elena.stocco@gmail.com (E.S.); silvia.barbon@unipd.it (S.B.); cinzia.tortorella@unipd.it (C.T.); veronica.macchi@unipd.it (V.M.); raffaele.decaro@unipd.it (R.D.C.)

**Keywords:** carotid body, growth factors, BDNF, GDNF, VEGF, receptors, development, hypoxia, hyperoxia, receptor–receptor interactions, Parkinson’s disease

## Abstract

The carotid body may undergo plasticity changes during development/ageing and in response to environmental (hypoxia and hyperoxia), metabolic, and inflammatory stimuli. The different cell types of the carotid body express a wide series of growth factors and corresponding receptors, which play a role in the modulation of carotid body function and plasticity. In particular, type I cells express nerve growth factor, brain-derived neurotrophic factor, neurotrophin 3, glial cell line-derived neurotrophic factor, ciliary neurotrophic factor, insulin-like-growth factor-I and -II, basic fibroblast growth factor, epidermal growth factor, transforming growth factor-α and -β, interleukin-1β and -6, tumor necrosis factor-α, vascular endothelial growth factor, and endothelin-1. Many specific growth factor receptors have been identified in type I cells, indicating autocrine/paracrine effects. Type II cells may also produce growth factors and express corresponding receptors. Future research will have to consider growth factors in further experimental models of cardiovascular, metabolic, and inflammatory diseases and in human (normal and pathologic) samples. From a methodological point of view, microarray and/or proteomic approaches would permit contemporary analyses of large groups of growth factors. The eventual identification of physical interactions between receptors of different growth factors and/or neuromodulators could also add insights regarding functional interactions between different trophic mechanisms.

## 1. Introduction

The carotid body (CB) is the main structure devoted to peripheral arterial chemoreception. In the recent years, many studies have reported the involvement of the CB also in other functions, such as sensing of metabolic (e.g., Sacramento et al. [1] and Kim and Polotsky [2]) and immunologic (e.g., Porzionato et al. [3]) signals.

The CB is composed of lobules containing type I (or chief or glomus) cells, positive for tyrosine hydroxylase (TH), and type II (or sustentacular) cells, positive for glial fibrillary acidic protein. Type I cells produce many different neurotransmitters/neuromodulators and are considered the real chemoreceptive structures, whereas type II cells are usually considered supportive cells, enveloping type I cells. It has been stressed, however, that type II cells probably interact with type I cells for coordination of chemosensory transduction [4,5] and that type II cells are the stem cell precursors for type I cells [6]. The sensory innervation of the CB is mainly given by the carotid sinus nerve (reviewed in Porzionato et al. [7]), a glossopharyngeal branch with sensitive neurons in the petrosal ganglion. Sympathetic and parasympathetic innervation is also present. The CB also shows a complex microvascularization, being one of the structures with the highest blood flow per gram of tissue; local modifications in blood flow may modify chemoreceptor discharge.

The CB structure and function may undergo plastic changes during development (e.g., De Caro et al. [8]) and ageing, and in response to environmental stimuli, such as hypoxia, hyperoxia, and exposure to nicotine. From a clinical point of view, the CB, and its structural/functional modifications in response to internal/external stimuli, may play a role in a wide series of pathological conditions, such as respiratory diseases, hypertension, heart failure, diabetes, obesity, sepsis, and others.

**Figure 1:**
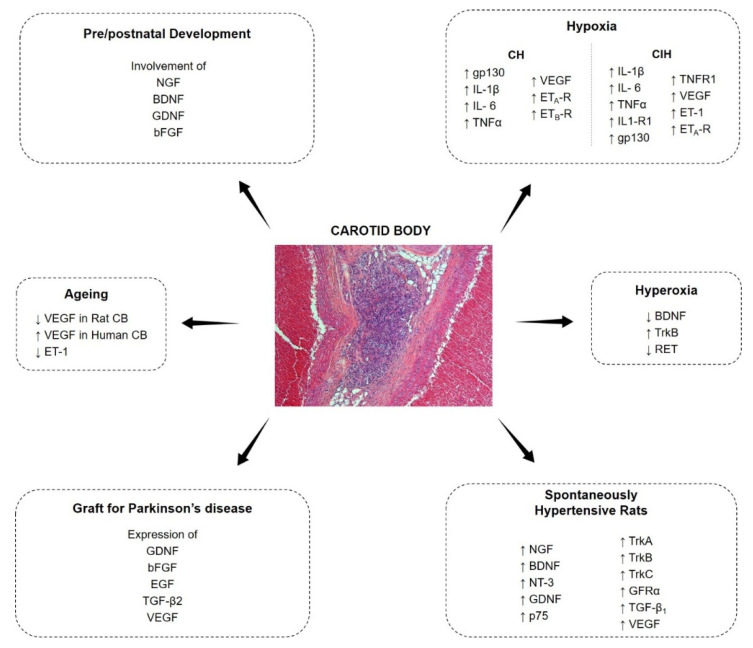
Expression and modulation of growth factors in the carotid body.

The CB cells express a wide series of growth factors and correspondent receptors; growth factors are known to play a role in the development of most plastic modifications of the CB (Figure 1). In the present review paper, an update of the most recent literature will be given about growth factors in the CB.

## 2. Nerve Growth Factor Family

The nerve growth factor (NGF) family, also referred to as neurotrophins, includes nerve growth factor (NGF), brain-derived neurotrophic factor (BDNF), neurotrophin (NT)-3, and NT-4/5 (also known NT-4). p75 low-affinity NGF receptor binds all the above neuronal growth factors. Tyrosine receptor kinase A (TrkA), TrkB, and TrkC are high-affinity receptors which preferably bind NGF, BDNF/NT-4/5, and NT-3, respectively (e.g., Barbacid [9]).

### 2.1. Nerve Growth Factor

Aloe and Levi Montalcini [10] demonstrated that anti-NGF antibodies exposure in rat fetal and early postnatal life caused CB hypoplasia. In the same years, Lawson [11] reported that NGF can determine the conversion of human CB cells to neuronoid-like cells with extensive dendritic processes. Further, in vitro data on NGF contribution to increased Na^+^ channel expression/density and/or Na^+^ and K^+^ currents was also reported [12,13,14,15]. On the contrary, late fetal and neonatal glomus cells were reported to survive in vitro in the absence of NGF or even in the presence of anti-NGF, and NGF was found not to have any effect on survival and DNA synthesis of type I cells [16,17,18,19]. Assumptions suggesting that CB cells respond to NGF after fibroblast growth factor (FGF) exposure were also reported by Zhong and Nurse [15].

Following the first studies on the NGF in the CB, Atanasova and Lazarov [20] evaluated NGF, TrKA, and p75 expression by immunohistochemistry (followed by immunostaining quantification) in the CB of spontaneously hypertensive rats versus normotensive ones. According to the study results, a stronger NGF expression and a moderate NGF expression were detected in type I cells and in a subset of type II cells, respectively, in case of hypertension. The same expression pattern was observed also for NGF receptors, TrkA, and p75 (Tables 1 and 2).

### 2.2. Brain-Derived Neurotrophic Factor

The BDNF was initially identified by Hertzberg et al. [21] and Brady et al. [22] in the rat CB by RT-PCR, Southern blotting [21], and in situ hybridization [22] approaches. In addition, nerve fibers showed immunopositivity to the factor [22] (Table 1).

Izal-Azcárate et al. [23] showed by double immunofluorescence the BDNF expression in type I cells where it has a role in the CB prenatal and postnatal maturation/survival/plasticity and innervation, by autocrine and paracrine signaling [24,25,26,27]. In fact, BDNF null mutant mice show a reduction in peripheral O_2_ chemosensitivity, with altered breath rhythm in normoxia, breath depression, and apneas [25]. The reduction or lack in BDNF also determines an altered CB maturation, and a loss in chemoafferent dopaminergic neurons in the nodose petrosal ganglion [25].

The effects of hyperoxia on BDNF expression in the CB were investigated by Dmitrieff et al. [25] and Chavez-Valdez et al. [28]. Pregnant rats (24–48 h prior to delivery) were exposed to hyperoxia in a chamber flushed with 60% O_2_ and < 0.4% CO_2_ and then the litters were maintained at the same gas concentration for 3 days after birth [25] or 7/14 days, followed by a further period of room air exposure (until day 9–10 or day 18–19 from birth, respectively) [28]. For hyperoxic pups, both Dmitrieff et al. [25] and Chavez-Valdez et al. [28] observed CB hypoplasia. According to Dmitrieff et al. [25], this modification was indirectly related to changes in the BDNF. The authors mainly focused on the BDNF receptor TrkB whose high mRNA expression level (quantitative RT-PCR) in hyperoxia not corresponding to variation in TrkB protein expression (Western Blot analysis) was in turn associated with low BDNF levels. By real-time qRT-PCR, Chavez-Valdez et al. [28] also demonstrated a selective reduction in BDNF gene expression and protein expression after 7 days of hyperoxia. In addition, the exposure to high O_2_ levels for 14 days was also associated with the irreversible decrease in the chemoafferents’ number in the petrosal ganglion [28].

In the CB of spontaneously hypertensive rats, Atanasova and Lazarov [20] observed higher expression levels of BDNF (together with NGF, NT-3, and GDNF and the related receptors p75, TrkA, TrkB, and TrkC) by immunohistochemical approach (Table 2).

Bairam et al. [29] analyzed long-term consequences of neonatal caffeine treatment (NCT) on BDNF and TrkB expression in the CB of male adult rats by RT-PCR and Western blot. In fact, it is known that administration of NCT to adult male rats increases the CB expression of A_2A_ receptor which (together with A_2A_ receptor agonists) can stimulate the synthesis of BDNF and TrkB. The animal model was developed by daily administration (oral gavage) of caffeine citrate, 15 mg/kg, from postnatal day 3 to 12 (0.05 mL/10 g body weight). The study results demonstrated that a) neither BDNF nor TrkB were affected by NCT in both male and female rats and b) changes in A_2A_ neurotransmission due to NCT did not alter BDNF levels and TrkB in the CB and nucleus tractus solitarius. Although NCT-mediated changes in A_2A_ neurotransmission showed not to alter TrkB levels, further investigation should aim to clarify the occurrence of a possible modification/alteration of the TrkB phosphorylated form, potentially affecting respiration.

Bavis et al. [27] considered the role of TrkB in the postnatal development of rat CB. The authors administered to rat pups a nonselective and a selective TrkB receptor inhibitor (K252a and ANA-12, respectively) for chronic and acute studies; both treatments resulted in a significant CB volume reduction (−35% and −18% with K252a and ANA-12, respectively). These findings further supported the hypothesis that endogenous BDNF normally sustains postnatal cell proliferation/survival by an autocrine and paracrine mechanism. Thus, an inadequate BDNF/TrkB signaling may be responsible of impaired CB development and CB-mediated reflexes, causing respiratory disfunction.

The evaluation of chronic hyperoxia on postnatal CB development was the research target of Dmitrieff et al. [25]; by mRNA expression studies and Western blot analysis, the authors demonstrated that a reduced signaling mediated by the TrkB receptors may have a role in CB hypoplasia.

Atanasova and Lazarov [20] evaluated the expression of various growth factors in a rat model of hypertension. In particular, by immunohistochemistry, they found high TrkB expression in both type I and type II cells of hypertensive rats. Thus, the authors suggested a role for growth factors in the CB plasticity in hypertension.

### 2.3. Neurotrophin-3 and Neurotrophin-4/5

Pello et al. [30] were unable to demonstrate the NT-3 presence on the CB of adult human subjects by immunohistochemistry. The immunopositivity for NT-3 was later demonstrated by Atanasova and Lazarov [20] in rat CB (Table 1). TrkC and p75 receptor were also identified by Atanasova and Lazarov [20] in rat CB type I and type II cells, respectively by immunohistochemistry.

Till date, no data on the NT-4/5 in the CB are available.

According to in vitro and in vivo data, the treatment with NT-4 (and BDNF) but not with NT-3 (and NGF) can replace the neurotrophic maintenance of the rat CB on chemoafferent neurons [20].

## 3. Glial Cell Line-Derived Neurotrophic Factor Family of Ligands

Glial cell line-derived neurotrophic factor (GDNF) family ligands (GFLs) are recognized to have a leading role in the development and function of the nervous system. This superfamily is represented by four members: the GDNF, neurturin (NRTN), persephin (PSPN), and artemin (ARTN), showing potent effects on different neuronal cell types, including the derivatives of the neural crest, like the CB cells [24,31].

Among the GFL, the GDNF majorly attracted the researchers’ interests. It is the ligand of the GDNF-associated glycosyl-phosphatidylinositol-anchored coreceptor (GFRα1) and of the Ret tyrosine kinase (RET) receptor. NRTN, ARTN, and PSPN specifically bind to the ligand-binding components GRFα2, GRFα3, and GRFα4 of the RET receptor, respectively [24].

### 3.1. Glial-Derived Neurotrophic Factor

The GDNF was identified in the rat CB [32,33,34,35,36] and in the mouse CB [36,37,38,39] by different methodological approaches including in situ hybridization, ELISA, RT-PCR, immunohistochemistry, and microarray analysis [32,33,34,35,36,37]. Only X-gal staining allowed for a more precise localization at the type I cells [34,36] (Table 3).

Among the CB secreted neurotrophic factors, the “dopaminotrophic” GDNF is one the most intensely investigated. Together with BDNF, it has a critical role in the prenatal/postnatal development and in vivo innervation of the CB. Mutant mice with null-expression of BDNF and GDNF showed depressed/irregular normoxic breathing and apneas as well as reduced number of dopaminergic neurons in the nodose-petrosal ganglion [25,37,40]. In case of hyperoxia, the GDNF was suggested to have a role mediating CB plasticity. In this condition, despite any variation in mRNA expression (qRT-PCR) or protein content (ELISA) (exposure time: 3 days), quantitative RT-PCR analysis showed a 34% reduction for mRNA GDNF receptor RET level, suggesting a hyperoxia-induced modification on GDNF signaling pathway [25].

Type I cells are reported to be one of the few areas in the nervous system expressing high levels of GDNF in adult life. Expression is species-independent as mouse, rat, swine, and human showed comparable expression-range levels [41]. Later, Ortega-Sáenz et al. [42,43] confirmed these findings and showed in human and rat CB that there was no age- or sex-related quantitative difference in GDNF content by PCR and ELISA. Similarly, no differences were also found in the numbers of progenitor and glomus cells. The collected data allowed to speculate on the GDNF neuroprotective role, thus suggesting its potential in promoting CB cells survival during ageing and its possible role in the use of CB grafts as Parkinson’s disease (PD) therapy to counteract neurological disorders [41].

Conversely, a lack of GDNF expression was observed in the superior cervical ganglion (SCG) [42,43] with a significant difference in GDNF protein amount between CB and SCG of both humans and rats. In GDNF knockout mice, the suppression of GDNF expression in adult life is reported to be responsible of extensive catecholaminergic neuronal death [41]. These evidences support the idea that the GDNF may have an active role in promoting the survival of CB cells during ageing by an autocrine/paracrine mechanism [42]. For long time, GFLs signaling was thought to be mediated by the receptors RET and GFRα. Conversely, to date, it has been suggested that the situation could be more complex than originally assumed, as suggested by discrepant expressions of RET and GFRα. In particular, according to Paratcha et al. [31], additional signal transducing GDNF receptors may exist and the neural cell adhesion molecule (NCAM) may be included among these. By immunostaining, Porzionato et al. [44] showed NCAM-positive type I cells in human CB samples and suggested a NCAM role in regulating adhesion between type I cells. Moreover, as NCAMs have a role in neural mechanisms of differentiation, survival, and cell plasticity, they may be active in the development/differentiation process of the CB including cellular/molecular changes related to chronic hypoxia (CH). Conversely, NCAM was not detected in type II cells. On the basis of the above considerations, NCAM in type I cells could be also implicated in trophic actions of GDNF.

Atanasova and Lazarov [20] also found significantly higher values of immunostaining for GDNF and the GFRα in type I cells of hypertensive rats than normotensive ones (Table 4). Conversely, no GDNF and GFRα positive type II cells were detected. According to these data, GDNF, together with other neurotrophins (NGF, BDNF, and NT-3), may have a role in both the onset and maintenance of hypertension, probably by the chemoreceptor-related stimulation of the sympathetic activity.

### 3.2. Neurturin, Persephin, and Artemin

According to RT-PCR analysis, ARTN was found to be expressed in the rat CB, while NRTN resulted to be barely detectable [35]. The expression of GRFα2-3 in rat CB was demonstrated by RT-PCR. Further, GRFα2 expression was also confirmed in type I cells by immunohistochemistry and in type II cells by confocal analysis. GRFα3 localization has not been recognized yet. Currently, no data about GRFα4 and PSPN are available [35].

According to an in vitro study by Leitner et al. [35], a cocktail based on ARTN, NRTN, PSPN, and GDNF was unable to promote survival/proliferation of embryonic-day 17 rat CB cultures, even though ARTN, NRTN, and GDNF sustained neurite outgrowth in type I cells.

## 4. Ciliary Neurotrophic Factor Family

The ciliary neurotrophic factor family includes the ciliary neurotrophic factor (CNTF), the leukemia inhibitory factor (LIF), oncostatin M, granulocyte colony-stimulating factor, IL-11, and cardiotrophin. The receptors belonging to this family exert their function by the Janus kinase/signal transduction and activator of transcription (JAK/STAT) signaling pathway [24].

CNTF expression was recognized in the type I cells of adult rats by double immunofluorescence [23]. Its function is mediated by the CNTF receptor composed of an extracellular subunit (i.e., CNTFRα subunit) and two transmembrane proteins (LIF receptor β—LIFR β; glycoprotein 130—gp130) [47]. The LIF receptor is a gp130-LIFR β heterodimer [48] (Table 5).

gp130 mRNA transcripts were detected in the rat CB by RT-PCR, whereas the protein was recognized in type I cell by Western blotting and double immunostaining approaches [49,50]. An upregulation of gp130 mRNA was detected in rat CB in case of CH [50].

No data about the production of the other CNTF family members by type I cells are reported in the literature.

## 5. Insulin and Insulin-Like Growth Factors

Insulin-like growth factors (IGFs) include the IGF-I and the IGF-II, which bind to the receptors IGF-IR and IGF-IIR, respectively. IGF-IR, a transmembrane glycoprotein endowed with tyrosine kinase activity, can also bind IGF-II but with lower affinity than IGF-I. Signal transduction is only mediated by the IGFR-IR; IGF-II binding to the IGF-IIR only modulates the amount of unbound IGF-II, without transducing signals [51].

IGF-I expression was reported in the adult rat type I cells trough double immunofluorescence [23], whereas, according to immunostaining data, IGF-II was detected in about 10% of the human type I cells [52], with an increase (from 20% to 60%) in case of CB tumors [52,53] (Table 5).

According to in vitro evidences, IGF-I increases rat CB cultures survival [54]; for IGF-II, no functional data are available.

Further studies are required to confirm IGF receptors in type I cells. On the other hand, the expression of insulin receptors in the rat CB was demonstrated by Western blot analysis [55], suggesting that IGF paracrine/autocrine effects could potentially be mediated by these receptors.

## 6. Fibroblast Growth Factors

Among the different forms of fibroblast growth factor (FGF1-22) currently identified in humans [56,57], only basic FGF (bFGF), corresponding to FGF2, was reported to be expressed in the CB. In particular, type I cells of rat [23,58] and human [59] tissue sections were found to exhibit strong immunoreactivity for bFGF. At the same time, high immunoreactivity for bFGF was detected also into dispersed cultures of P1 rat CB [58] as well as in paragangliomas [59], with tumors presenting lower staining intensity and percentages of positive cells in comparison with the CB [59]. Type II cells of rat CB also showed to be positive to bFGF immunostaining, although in a mean percentage lower than 50% [23] (Table 6).

Receptor for FGF (FGFR) was localized by immunohistochemistry into glomus cells of postnatal CB cultures, both in normoxic and hypoxic rats, and in the presence or absence of bFGF [58]. In the human CB, the majority of type I cells exhibited weak to moderate cytoplasmic immunoreactivity for FGFR1, with stronger staining intensity and higher percentage of positive cells detected in paragangliomas rather than CB [59] (Table 7).

In type I cells from E18 to E19 rat pups, treatment with bFGF for 2 days demonstrated to increase both inward Na^+^ and outward K^+^ currents, also after normalizing for the increase in cell size [15]. In addition, in cell cultures from CB of E17 to E19 rat pups, bFGF promoted survival and BrdU incorporation. Finally, in postnatal P1-P3 cultures, bFGF resulted to stimulate DNA synthesis without affecting cell survival [19]. In order to define the role of endogenous bFGF release in cell cycling and survival in postnatal CB cultures, a neutralizing antibody was administered to both normoxic and hypoxic rats, identifying no significant effects on DNA synthesis but significantly decreased survival of glomus cells [58]. Interestingly, bFGF induced neuronal differentiation in fetal rat glomus cells by producing neurite outgrowth and increasing neurofilament immunoreactivity, with no similar effects detected in postnatal cultures [19].

## 7. Epidermal Growth Factor/Transforming Growth Factor-α Family

The epidermal growth factor/transforming growth factor-α (EGF/TGF-α) family comprises a series of peptides including, besides EGF and TGF-α, amphiregulin, heparin-binding EGF, betacellulin, pox virus growth factor, cripto, and heregulin. These peptides share the capacity to bind to the EGF receptor (EGFR).

Type I cells of rat CB were proven to express EGF by double immunofluorescence analysis. In parallel, EGFR immunoreactivity was detected in both type I and II cells, with the latter ones showing positive labeling in less than 50% of cells. In addition, also TGF-α was immunohistochemically localized in type I cells of the rat CB [23] (Table 6 and Table 7).

## 8. Transforming Growth Factor-β and Related Molecules

The proinflammatory factor TGF-β is known to play a role in the regulation of extracellular matrix deposition by collagen synthesis. Related TGF-β homologs are morphogenetic proteins (BMPs), which are expressed in several regions of the embryonic brain and neural crest-derived tissues. Both TGF-β and BMPs bind to heteromeric transmembrane complexes consisting of receptor serine/threonine kinases, termed types I and II [64].

TGF-β1 expression was localized in the rat CB by immunohistochemistry, resulting to be upregulated in spontaneously hypertensive rats (SHR) and in SHR animals treated with the beta-blocker atenolol (Table 6) [63]. On the other hand, the treatment with the angiotensin-converting enzyme inhibitor ramipril limited factor upregulation in SHR rats [62,63]. Thus, the stimulation of TGF-β1 expression and extracellular matrix augmentation induced by high blood pressure seem to occur also in the CB by activation of the renin–angiotensin–aldosterone system.

Regarding BMPs, BMP2 expression was detected in the mouse CB, with lower positivity in A/J than in DBA/2J mice, indicating that this factor could produce phenotypic differences in morphology and neurotransmitter expression between the two strains [39] (Table 6).

To the best of our knowledge, no studies have been reported yet about the presence of the receptors for TGF-β1 and BMP homologs in the CB.

## 9. Inflammatory Cytokines

Inflammatory/immunological factors have been demonstrated to play a role in the physiology and plasticity of the CB. Glomus cells produce the proinflammatory cytokines interleukin (IL)-1β, IL-6, and tumor necrosis factor α (TNFα) with corresponding receptors, which regulate CB excitability, catecholamine release and/or chemoreceptor discharge (reviewed by Porzionato et al. [3,24]).

In particular, inflammatory cytokines (i.e., IL-1α, IL-1β, IL-6, and TNFα) were proved to be expressed into type I cells of the rat CB by RT-PCR [49,65,66], quantitative Real-Time PCR (qPCR) [67,68], amplified RNA (aRNA)/qPCR technology [69], RNA sequencing [68], ELISA [68], double immunofluorescence [49], and immunohistochemical studies [70,71,72,73] (Table 8).

Immunofluorescent double-labeling technique was used to colocalize IL-1β, IL-6, and TNFα and their receptors (IL-1R1, gp130, and TNFR1, respectively) with TH into glomus cells [50,65,66] (Table 9).

IL1-R1 was detected in the rat CB by RT-PCR, Western blot, and immunohistochemistry/double immunofluorescence [49,50,74]. Receptor expression was mainly localized into type I cells, with slight positivity found also in blood vessels, type II cells, and fibroblasts.

Messenger RNAs of TNFR1 and TNFR2 were also detected into rat CB by RT-PCR [49,65,66,70]. Double immunofluorescence identified TNFR1 in TH-positive type I cells [65,66], whereas TNFR2-immunoreactivity was localized surrounding CB clusters of TH-positive glomus cells [65] or into endothelial cells [70] (Table 9).

Apart from type I cells, in situ hybridization localized IL-6 expression also in type II cells [69], and IL-6 concentrations of the CB lysate were also measured with ELISA [75,76]. Regarding IL-6 receptors, IL-6Rα expression was demonstrated into type I cells of the rat CB by Western blot, double immunofluorescence, and in situ hybridization [50]. Moreover, gp130 mRNA and protein were detected in rat CB by RT-PCR [49,50,66] and Western blot [50], respectively. Notably, its localization into type I cells of both rat and human tissue sections was demonstrated by double immunofluorescence [49,50,66,76] (Table 8 and Table 9).

The effects of proinflammatory cytokines on the neurogenesis of CB were investigated for the first time by Xue and colleagues [77]. Exposure of rat CB to intermittent hypobaric hypoxia (IHH) promoted the phosphorylation of extracellular signal-regulated kinase (ERK) 1/2 (determining neuronal progenitor cell fate), as well as increased the expression of TH and nestin as a specific neuronal stem cell marker. Moreover, the intraperitoneal administration of IL-1β had an additive effect on IHH. According to these results, the treatment with IL-1β is suggested to increase the plasticity of CB, while ERK1/2 seems to play a role in neurogenic signaling in CB [77].

Chronic hypoxia was found to upregulate by >2-fold the expression of the proinflammatory cytokines IL-1β, IL-6, and TNFα into the rat glomus cells during the early phases of treatment (1–3 days). During long-term exposure (28 days), IL-6 remained elevated >5-fold, whereas expression of the other cytokines recovered to normal levels. Interestingly, the increased expression of IL-6 was observed not only in glomus cells but also in type II cells [69]. In addition, Liu and colleagues [69] demonstrated that CH promoted the recruitment of macrophages in the CB, supporting the role of immune elements and inflammatory cytokines in the development of altered CB structure and increased chemosensitivity in this pathologic condition. Subsequently, Liu and collaborators [67] collected experimental evidence supporting the hypothesis that CH-induced inflammation responses in the CB could be mediated by type-A endothelin (ET-A) receptors expressed by both chemosensory type I cells and immune cells.

The upregulation of IL-1β, IL-6, and TNFα was also observed under chronic intermittent hypoxia (CIH) conditions [66,71,72,73], while IL-6 expression resulted to be stimulated also by intermittent hypoxia/reoxygenation (IH/ROX) treatment, which simulates the pattern of hypoxic episodes seen in obstructive sleep apnea [75]. Besides cytokine upregulation, also corresponding receptors IL-1R1, gp130, and TNFR1 were found to be overexpressed in the lobules of chemosensitive glomus cells containing TH, after exposure of rat CB to CIH conditions (alteration of oxygen levels between 21% and 5% ± 0.5% per min, 60 cycles/h, 8 h/day) up to 7 days [66]. In addition, rat CB exposed to CIH (5% O_2_, 12 times/h per 8 h) for 7, 14, and 21 days showed a progressive increase in IL-1β, TNFα, and iNOS immunoreactivity over time. In CIH, Del Rio et al. [71] reported that overexpression of proinflammatory cytokines was not associated with immune cell invasion within the CB, but in a following work by Lam et al. [66] macrophage infiltration resulted to be significantly promoted by CIH conditions, showing local inflammation of the CB.

Exogenous cytokines were found to enhance the intracellular calcium response to acute hypoxia in the dissociated glomus cells of CIH-exposed CB [66]. Remarkably, inflammatory cytokines were also shown to play critical roles in mediating phenotypic adjustments in type I cells during sustained hypoxia. Liu and coworkers [78] exposed cultured type I cells to hypoxia for 24 h and to a cocktail of cytokines consisting of IL-1β, IL-6, and TNFα. Evaluation of hypoxia-induced intracellular Ca^2+^ responses showed that the exposure to cytokines plus hypoxia significantly increased cell excitability. Moreover, the authors demonstrated that cytokine effects are correlated to the upregulation of the transcription factor hypoxia inducible factor-1α (HIF-1α) and consequent overexpression of specific hypoxia-sensitive genes (i.e., adrenomedullin) in type I cells.

Interestingly, anti-inflammatory treatment with ibuprofen or dexamethasone showed to attenuate the oxidative stress and significantly reduce the upregulation of cytokine expression and immune cell infiltration in chronic intermittent hypoxia-exposed CB [66,69,72]. At the same time, ibuprofen administration to CIH-exposed rats failed to decrease the enhanced CB chemosensory reactivity to hypoxia [73]. Overall, these results confirmed that the upregulated expression of proinflammatory cytokines could mediate the local inflammation and functional alteration of the CB under CIH conditions [66] but seems not to be linked to the mechanisms underlying the potentiation of the CB chemosensory response to acute hypoxia [73].

The effect of exogenous cytokine administration to dissociated glomus cells was tested not only on CIH-exposed CB [66] but also on unstimulated chemoreceptor organ. An in vitro study by Fan and colleagues [79] investigated the effect of IL-6 on [Ca^2+^]_i_ and catecholamine (CA) secretion into rat CB cell cultures. After IL-6 administration, treated cells showed an increase in [Ca^2+^]_i_ determined by fluorometric measurements. In addition, amperometric analysis revealed that IL-6 application induced the release of catecholamine by glomus cells, with response abolished by the calcium channel blocker Cd^2+^. These data confirmed the CB ability to respond to proinflammatory cytokines, highlighting its role in inflammation sensing and transmission of such information to the brain [79].

Expression of proinflammatory cytokines by CB was also investigated in human samples obtained from surgical patients undergoing elective head and neck cancer surgery. CB slices exposed to sustained hypoxia for 1 h showed an increased release of IL-1β, as well as IL-4, IL-6, IL-8, and IL-10, measured by multiplex ELISA. In parallel, immunohistochemical studies confirmed that human type I cells also express IL-1R1 and interleukin 6 receptor (IL-6R) [76].

Lipopolysaccharide (LPS) administration in rats was found to evoke TLR4/MyD88-pathway activation in CB with consistent upregulation of TNFα and TNFR2 in TH-containing glomus cells [65]. Furthermore, endotoxemic rats subjected to bilateral section of the carotid sinus nerve exhibited altered plasma TNFα and immunomodulator (i.e., corticosterone, cortisol, and epinephrine) levels, increased multiorgan damage, and lower survival rates, confirming the protective role played by carotid chemo-/ baroreceptors in sepsis syndrome progression [80].

Contributing to the growing body of evidence which suggests that immune signaling can modulate CB function, a very recent study investigated the ex vivo treatment of rat CB with the damage-associated molecular patterns (DAMPs) and humoral factors of aseptic tissue injury. CB exposed to various DAMPs (i.e., all-thiol and disulfide HMGB1 and S100 A8/A9) demonstrated a significant increase in TNFα release. A similar effect was detected in CB incubated with conditioned blood plasma obtained from a rat model of aseptic injury. In addition, all-thiol HMGB1 resulted to upregulate some prominent proinflammatory markers, including IL-1α and IL-1β. Interestingly, conditioned plasma had a stronger effect on the regulation of CB transcriptome, leading to inhibition rather than stimulation of immune-related pathways. These findings supported for the first time the involvement of endogenous mediators of innate immunity in the modulation of CB function [68].

Besides LPS and DAMPs, also the growth factor-like lipid lysophosphatidic acid (LPA) is worth being mentioned among potential modulators of the CB in inflammatory conditions. In particular, preclinical studies on a rat model of asthma showed that the administration of LPA, at concentrations present in the blood following allergen/bradykinin challenge, stimulate the CB by activating G-protein coupled receptors (GPCRs), causing acute bronchoconstriction [81]. Moreover, this effect is counteracted by blocking this pathway with receptor antagonists. Thus, these data suggest the relation between CB-mediated bronchoconstriction and the inflammatory disease asthma.

## 10. Vascular Endothelial Growth Factor

Type I cells have been proved to express vascular endothelial growth factor (VEGF) as well as the VEGF receptors Flt-1 (VEGFR1) and Flk-1 (VEGFR2) (reviewed by Porzionato et al., [24]). VEGF acts on Flk-1 to regulate hyperplasia of the type I cells and promotes neovascularization by interaction with Fit-1 in endothelial cells [82].

With respect to our previous review paper, more recent studies confirmed that VEGF is expressed in type I cells of rat CB, as highlighted by double-immunostaining with TH [61], immunohistochemistry [83,84,85,86,87,88,89,90], and qRT-PCR for mRNA expression study [91]. This growth factor has also been localized in human CB by immunohistochemical studies [92,93]. VEGF concentrations into CB lysate was also determined by ELISA [75] (Table 10).

VEGFR1 and VEGFR2 immunoreactivities were detected into cell clusters in the rat CB [83,84] but more recent studies about their expression have not been developed so far.

During development and ageing, modifications in oxygen supply to tissues lead to critical adaptive responses, such as vascular remodeling. Considering that, changes of VEGF expression related to CB physiological denervation were assessed into senescent (22 months old) male Wistar rats compared to young (2–3 months old) animals. In particular, CB showed significant adaptation to ageing process by reductions in the sensitivity to stimuli and the decrease in VEGF release along with age. Moreover, age-related reduction in synaptic junctions was observed between chemoreceptor cells [88]. Conversely, studying human CB sampled at autopsy, a significant increase in VEGF immunoreactivity was detected in carotid tissues from children (mean age: 2 years) and aged subjects (mean age: 67.3 years) compared with young adults (mean age: 44.3 years). In parallel, nitric oxide synthase (NOS) immunoreactivity was higher in CB tissues from the children and young adult subjects compared with the old subjects, suggesting that oxygen-sensitive CB mechanisms are affected in an age-dependent manner [92].

Since hypoxia exposure is known to increase the CB microvascularization, in addition to number and size of glomus cells (e.g., Prabhakar et al. [82]), the regulation of VEGF expression has been widely investigated in relation to this type of stimulus [75,89,91,93]. Intermittent hypoxia/reoxygenation exposure in rabbits increased CB production of VEGF (measured by ELISA), suggesting the involvement of this growth factor in the adaptive pathway during IH/ROX challenges [75]. In male adult Sprague-Dawley rats exposed to CIH (5% O_2_, 12 times/h for 8 h/day in a 21 days period), VEGF immunoreactivity (VEGF-ir) and the size—but not the number—of the blood vessels into the CB significantly increased, while changes in CB volume or number of glomus cells were not observed. In addition, antioxidant treatment with ascorbic acid, which prevents the CB chemosensory potentiation induced by CIH, failed to block the vascular enlargement and the increase in VEGF-ir, suggesting that these effects are directly related to intermittent hypoxic conditions and not secondary to the oxidative stress [89]. More recently, the upregulation of VEGF, together with TH mRNAs, was demonstrated into CB obtained from rats reared under chronic hypobaric hypoxia (~60 kPa, equivalent to 4300 m) for 5–7 days [91]. Finally, significantly higher positivity to VEGF was found into CB of heroin-addicted subjects compared to subjects who died for traumatic causes, possibly due to the hypoxic stimuli related to heroin intoxication [93].

Not only hypoxic conditions but also hypertension has been shown to upregulate VEGF expression in the rat CB [90]. Administration of hydrochloride NG-nitro-methyl ester-L-arginine (L-NAME) for 6 weeks to male Wistar rats is known to induce hypertension, causing significant morphological changes in the CB (i.e., higher size dimensions; hypertrophy, and cytoplasmic vacuolation of type I cells and increase in elastic fibers, proteoglycans, and collagen fibers). According to immunohistochemical analyses, VEGF was also upregulated by L-NAME treatment. At the same time, VEGF was randomly distributed throughout glomus cells and blood vessels in the CB of this experimental model of hypertension, whereas it was mainly expressed by glomus cells in normal conditions [90].

## 11. Endothelins

Endothelin-1 (ET-1) and its receptors were previously documented to be either undetectable or expressed at low levels in adult normal CB, being significantly upregulated by CH [24,82]. In particular, ET-1 has been detected not only into endothelial cells of CB blood vessels by immunohistochemistry and immunoelectron microscopy [94,95] but also into glomus cells of the CB of rats [87,96,97,98] and cats [99,100,101,102] by immunohistochemical analysis (Table 11).

Consistent with earlier reports, immunocytochemistry identified ET-1 expression in many glomus cells of neonatal carotid bodies, as evidenced by colocalization with TH [103]. The mRNA expression of ET-1 and its receptors, ET_A_-R and ET_B_-R, into rat CB was demonstrated by both RT-PCR and qPCR [103,104,105]. Moreover, specific genes belonging to the ET-1 signaling pathway were detected into primary cultures of rat CB cells by cDNA Expression Array [106]. ELISA and enzyme immunoassay (EIA) allowed to determine ET-1 concentrations into rabbit CB lysates, as well as peptide content and release from rat CB [75,103,105]. ET-1 immunohistochemical localization into the rat CB was performed [71,107] also in combination with protein quantification, demonstrating that ET-1 was ubiquitously expressed in the blood vessels and CB parenchyma [107]. Double-label immunofluorescence studies demonstrated the colocalization of both ET-1 and ET_A_ receptors with TH into O_2_-sensitive type I cells [67,105] (Table 11).

In the rat and cat CB, ET_A_ receptor mRNA and protein expressions were firstly identified in type I cells by in situ hybridization, Western blot, and immunohistochemistry [96,97,98,101,102]. Moreover, ET_B_ receptor was identified in the cat CB by Western blot and into the cytoplasm of type I cells by immunohistochemistry [101,102]. More recently, the expression of ET_A_ and ET_B_ receptors into rat CB homogenates and CB cultured cells was detected by Western blot and immunoblotting analysis, respectively [106,108] (Table 12).

Hypoxia was proved to strongly increase the expression of ET-1, which plays a pivotal role in cellular adaptation to hypoxic conditions. ET-1 signaling pathway appeared to be differentially expressed in rat CB cultured cells under chronic hypoxia [106]. Changes on ET-1 level in the CB were explored after different protocols of intermittent IH/ROX exposure in rabbits. ET-1 expression resulted to be increased, but then decreased, by growing IH frequencies [75]. CIH upregulated ET-1 expression into glomus cells without significantly altering preproendothelin-1 mRNA levels. Interestingly, the activity of endothelin-converting enzyme (ECE) increased along with ET-1 upregulation, and ET-1 release from CIH-treated CB was also found to be enhanced [105]. Increased ET-1 expression during CIH was also associated to upregulated hypoxia-induced factor-1α (HIF-1α) into the CB, as well as to augmented HIF-1α and ET-1 levels into the plasma of treated animals [104].

The effect of ageing on ET-1 expression has been addressed by Di Giulio et al. [107], who reported under normoxic state, lower levels of expression in old rats (24 months old) with respect to young animals (3 months old). Exposure to CIH seemed to significantly increase ET-1 immunoreactivity in rat CB of both young and old animals, although its stimulation was shown to be dampened in the aged carotid bodies. This suggests that ET-1 may have a role in age-related changes of CB function [107].

Unlike the adult rat CB, IH was found to exert no significant effect either on ET-1 expression or pre-pro-ET-1 mRNA levels in neonatal CB, probably due to high basal levels of ET-1 expression detected into rat pups. At the same time, IH increased basal release of ET-1 from CB and upregulated ET_A_ but not ET_B_ mRNA expression [103]. Reports on time course studies investigating CIH-mediated effects on ET-1 expression in the rat CB demonstrated that intermittent hypoxia produced a transient increased of ET-1 immunoreactivity by about twofold, whereas ET-1 plasma levels remained unchanged. According to these findings, ET-1 may contribute to the enhanced CB chemosensory excitability during the early phase of the intermittent hypoxic challenge [71].

Ex vivo studies on the effects of exogenous ET-1 showed that femtomolar concentration augmented chemosensory response in IH-treated neonatal CB, whereas picomolar concentration was needed to obtain the same effect in normoxic CB [103]. Similarly, the infusion of ET-1 to CIH-exposed rats resulted in a substantial increase in carotid sinus nerve activity [108,109], with a significant reduction in case of prior administration of MK-801, an antagonist of the N-methyl-D-aspartate (NMDA) glutamate receptor [109]. Furthermore, phospholipase C, protein kinase C, and p38MAPK signals were also found to be involved in ET-1-enhanced carotid sinus nerve activity under CIH conditions, with calcium influx also contributing to this hypoxic sensory response [108].

The paracrine effects of ET-1 on glomus cells [110] and type II cells [111] were investigated by ratiometric Ca^2+^ imaging. Intriguingly, different results were reported for type I and type II cells, although in different studies. ET-1 produced either no or very weak increase in [Ca^2+^] into CB glomus cells at a concentration of 10 nM [110]. Conversely, lower concentrations of ET-1 (1 nM) induced robust intracellular Ca^2+^ responses in subpopulations of type II cells [111].

Recently, the possible effects of chronic exposure to hypoxia on the differentiation of CB neural stem cells (NSCs) into mature glomus cells have been investigated. To this end, CB stem cells and mature type I cells were isolated from rats and mice maintained in normoxic condition or chronically exposed to hypoxic environment (10% or 14% O_2_). In vitro cell investigations revealed that hypoxia seems not to affect CB NSC proliferation directly. Conversely, hypoxic conditions induce the upregulation of ET-1 expression/secretion by mature glomus cells, which establish synaptic-like contacts with stem cells expressing endothelin receptors, and so regulate their growth. In addition, the inhibition of glomus cell transmitter release or their selective destruction significatively reduce NSC growth during hypoxia, showing that stem cells are under the direct ‘‘synaptic’’ control of the mature O_2_-sensitive cells. This suggests that glomus cells not only acutely activate the respiratory center but also induce NSC-dependent CB hypertrophy necessary for acclimatization to chronic hypoxemia. [112].

Concerning ET-1 receptors, ET_A_-R mRNA expression into neonatal CB was demonstrated to be about 3.5-fold higher in IH rather than normoxic conditions [103,105]. This result was confirmed also by protein expression studies on ET_A_-R into adult rat CB exposed to CIH [108]. Accordingly, the increase in ET_A_ mRNA and protein was not seen in CB treated with receptor antagonist [103,105,108].

On the other hand, conflicting findings have been reported about ET_B_-R. After CB exposure to CIH treatment, this receptor was not upregulated in terms of mRNA [103] or protein [108].

Following CH, ET_A_-R was not only overexpressed into type I cells but also detected in resident and invasive CD45^+^ immune cells in the tissue surrounding chemosensory cell lobules [67]. On the other hand, CH also increased ET_B_-R protein expression into cultured CB cells [106]. According to immunofluorescence and quantitative PCR studies, concurrent treatment with the ET_A/B_ receptors antagonist (bosentan) blocked CH-induced macrophage invasion and the upregulation of pro-inflammatory cytokines and monocyte chemoattractant protein-1 (MCP-1). This supports the hypothesis that the inflammatory state induced by CH leads to the upregulation and release of ET-1 from type I cells. ET-1 may then act in an autocrine/paracrine mechanism via ET_A_-R on chemosensory type I cells and immune cells to promote an inflammatory response [67].

## 12. Platelet-Derived Growth Factor

Being firstly recognized as a mitogen for fibroblasts present in human serum and in alpha granules of platelets, platelet-derived growth factor (PDGF) was demonstrated to act also on glial cells and neurons, playing a role in cell proliferation during embryogenesis [24]. Trophic effects of this factor (formed by dimers of A and B subunits: PDGF-AA, -AB, and -BB) are mediated by PDGF receptor-a (PDGFR-a) and PDGF receptor-b (PDGFR-b) [113].

In the CB of rats, both type I and II cells demonstrated immunoreactivity to PDGFR-a, even though less than 50% of type II cells were positively labeled [23] (Table 7).

To date, data about PDGF expression CB cells are still missing, except for reported lack of PDGF immunoreactivity in human paragangliomas [114].

Thus, it might be worth investigating the possible role of PDGF in the CB, also considering the effect of hypoxic stimuli.

## 13. Receptor–Receptor Interactions involving Growth Factors Receptors

Receptors are generally depicted as isolated/integral membrane proteins located at the lipid bilayer where they mediate signal transduction in a linear stepwise manner. However, since the 1980s, the pioneering studies by Agnati et al. [115,116] and Fuxe et al. [117] on GPCR monomers demonstrated the existence of structural interactions between the above macromolecules. Such interactions were defined as receptor–receptor interactions (RRI), where the term RRI was adopted to emphasize the intrinsic nature of the synergy, requiring a direct physical contact between the receptors. Hence, each macromolecule involved in signal recognition/transduction can be part of a more complex and articulated system thus leading to the formation of dimers or high-order oligomers at the cell membrane level. Changes in the dynamic monomer/oligomer equilibrium are presumably correlated to functional implications [118,119,120].

In the CB, many evidences in the literature theoretically support the possibility of RRIs between different receptor types in type I and type II cells [121]; CB GPCRs are supposed to combine and form homo-/heterocomplexes responsible of a dynamic monomer/oligomer equilibrium with potential effects on chemoreception, neuromodulation, and tissue plasticity [119]. However, experimental studies empirically demonstrating the existence of such di-/oligomerizations are still scant in the CB [122,123], probably due to difficulties in the methodological approach.

For RRIs, two prerequisites are mandatory: (a) colocalization in “close proximity” (i.e., minus than 10 nm) of the receptors and (b) detectable biochemical/functional change in one receptor provoked by the binding of a ligand to another receptor [7]. As these associations are responsible of structural/functional mechanisms finely regulating cell fate and function, they undoubtedly represent an interesting field of investigation.

As previously discussed by Porzionato et al. [24] and considered above, the signal cascades induced by the growth factors in the CB are mediated by several growth factor receptors, including p75^LNGFR^, TrkA, TrkB, RET, and GDNF family receptors alpha1-3, RET, gp130, IL-6Rα, EGFR, FGFR1, IL1-R1, TNFR1, VEGFR1 and 2, ET_A_, and ET_B_ receptors, and PDGFR-a. According to the previous assumption on the GPCRs and specific experimental evidence in other anatomical districts, the occurrence of interactions between growth factor receptors in the CB could be a possible event too. Hence, to further support this hypothesis, their behavior in other in vitro/in vivo models can be considered.

The superfamily of receptor tyrosine kinases (RTKs) is involved in signaling mechanism regulating both cell proliferation and differentiation by the interaction with a wide array of growth factors including EGF, NGF, PDGF, VEGF, FGF, and IGF [124,125]. The RTK activity is based on association in the plasma membrane thus activating many intracellular signaling cascades (e.g., MAPK, PI3K, PKC, and STAT pathways) [126]. The RTKs readily form homodimers, however, they can also participate in heterointeractions with other RTKs. Heterodimerization between RTKs is believed to determine signal amplification and diversification, compared to homodimers. However, the extent of RTKs heterodimerization and partners of such heterointeractions are unknown [124].

NGF signals by binding TrkA and p75^NTR^; despite both receptors can function independently by interacting with NGF, the formation of a TrkA-p75^NTR^ heterodimer was demonstrated several years ago. To date, the precise mechanistic basis of this interaction remains uncertain; however, physicochemical modelling approaches suggest that p75^NTR^ binding to TrkA may induce a conformational change in this latter, increasing NGF affinity for TrkA [127,128].

Di Palma et al. [129] demonstrated by in situ proximity ligation assay (PLA) that TrkB can form heterogeneously distributed A_2_AR-TrkB heteroreceptor complexes in the rat dorsal hippocampus; this interaction seems to modulate the BDNF action on hippocampal plasticity resulting impaired upon ageing.

CNTF, after the interaction with the CNTFR (i.e., specific, nonsignaling receptor subunit), induces the formation of gp130-leukemia inhibitory factor receptor (LIFR) heterodimers [47,130,131]. Moreover, De Serio et al. [131] also demonstrated by immunoprecipitation that CNTF can also assemble a hexameric receptor complex made of two CNTF, two CNTFR, one gp130, and one LIFR molecule. As these evidences appear to be in contrast with Schuster et al. [132,133], further investigations are required.

ET_A_ and ET_B_ receptors can form heterodimers as demonstrated by FRET, coimmunoprecipitation, and calcium mobilization [121,134,135,136,137,138]; however, the signaling consequences of the ET_A_ and ET_B_ heterodimerization still remain poorly understood [121,136].

To date, limitations in experimental methods are responsible of a poor characterization of heterodimers when compared to homodimers. Integrating different biochemical and biophysical approaches such as FRET/BRET, PLA, radioligand binding assays, atomic force microscopy, and physicochemical modelling may represent the keystone for the research on this “new” intriguing field with also significant implications in the physiopathology of the CB.

## 14. The Trophic Role of CB Grafting in Parkinson’s Disease

Strategies involving cell-based therapies are gaining increasing interest in the treatment of PD, with the aim to reduce or replace neurons loss in the substantia nigra by the implant of healthy dopaminergic cells. Over the years, different types of cell grafts have been investigated, including human fetal brain cells, porcine dopaminergic cells, embryonic stem cells, neural stem cells, and genetically modified cells [139]. Encouraging results have been obtained by in vitro investigations, preclinical studies and clinical trials, with higher therapeutic efficacy demonstrated in animal models than in PD patients [46]. In this context, CB cells highly attracted the researchers’ attention for a possible clinical application in PD [45,46,140]. The therapeutic effect of intracerebral CB graft was initially attributed to the local release of dopamine by Type I cells. More recent studies pointed out that grafted CB cells may more likely act by the secretion of neurotrophic factors to improve the therapy outcomes, actively counteracting dopaminergic neurons degeneration [45,46,140].

A preclinical study on a chronic 1-methyl-4-phenyl-1,2,3,6,-tetahydropyridine (MPTP) mouse PD model treated with unilateral intra-striatal CB grafts showed a behavioral amelioration, together with histological evidences of significant recovery, as also proved by stereological quantification of dopaminergic substantia nigra neurons versus the shamed-grafted side [46]. Despite a possible dose-dependent effect may exist, related to both implant volume and/or GDNF expression levels in the grafts, the results highlighted that CB grafting determines neuronal protection and sprouting of dopaminergic fibers with activation of nigrostriatal neurons [46]. In parallel, also CB cografts were validated. The CB aggregates showed a synergic effect when grafted with a cell suspension from the fetal ventral mesencephalon (VM) in a 6-hydroxydopamine (6-OHDA) maximal unilateral DA dopaminergic denervation. Specifically, ELISA assay showed significantly higher GDNF content in VM + CB suspensions than in VM alone suspension cells. The in vitro data were further confirmed by functional cylinder test. However, to observe any improvement induced by CB grafts after 6-OHDA injection, a partial preservation of the nigrostriatal pathway was necessary. In case of complete denervation (i.e., long-term 6-OHDA), the improvements by CB cells were absent [45].

Together with GDNF, changes in the expression of bFGF and TGF-β2 in parkinsonian rats grafted with CB glomus cells were considered [60]. Working on the 6-OHDA rat model, glomus cells of the CB were transplanted into the lesioned striatum, evaluating bFGF and TGF-β2 expression at different time points after graft (i.e., 2, 4, and 12 weeks). Since endogenous expression and release of trophic factors by grafted glomus cells, as well as by the surrounding host tissue may promote graft survival and integration, administration of exogenous bFGF and TGF-β2 could improve eventual beneficial effects. Considering that, immunohistochemical studies on tissue explants were performed to localize bFGF and TGF-β2 expression in intrastriatally placed glomus cell grafts. Two weeks after transplantation, few bFGF-immunoreactive cells were localized in striatum near the grafts, with weak bFGF expression. At 4 weeks, cell graft and its surrounding area showed increased immunoreactive elements, indicating that the transplanted dopaminergic neurons and astrocytes at the graft–host interface expressed bFGF. Twelve weeks after graft, bFGF expression decreased again to the level observed at the first time point. Notably, both type I and type II transplanted glomus cells expressed bFGF at the same time course. Conversely, location of glial bFGF expression was not observed in the striatum surrounding the graft. These findings suggested that the expression of bFGF after transplantation could assure for better survival rates of dopaminergic grafts, so bFGF producing glomus cells of the CB may represent a valid grafting source for PD [60].

In parallel, TGF-β2 immunoreactivity was localized in the intrastriatal grafts, at the graft–host interface and in type I glomus cells within the graft 4 weeks after transplantation, with no labeling observed on striatal neurons at any time point investigated (Table 6). This expression pattern suggested that TGF-β2 survival-promotion effects are limited to specific subpopulations of dopaminergic neurons projecting to different target cells that permit access to the trophic factor [60].

Interestingly, preclinical neurogenesis researches considered the impact of CB-derived growth factors on migration and differentiation of neural progenitors in the subventricular zone/olfactory bulb (SVZ/OB)-system of hemiparkinsonian rats receiving intrastriatal CB graft. As expected, grafted glomus cells were found to express a variety of growth factors, including EGF. Based on that, the growth factors produced by grafted glomus cells were suggested to promote the differentiation of neural progenitors in the SVZ/OB-system, recognizing a possible role for CB cell transplantation in the self-repair of brain degenerative disfunctions [61]. Finally, VEGF expression was also demonstrated in intrastriatal grafts of glomus cells in rat models of PD, further supporting a neurogenic role of the CB graft in the repair of brain degenerative disfunctions [61].

## 15. Conclusions

In the present paper, we gave a wide survey of literature data about expression of growth factors and receptors in the different cell types of CB. Many of these growth factors have been studied with specific reference to environmental stimuli or in vivo experimental models of various pathologies (hypertension, diabetes, and obesity). Some aspects, however, have not yet been adequately considered at the moment and will need to be addressed in the future. For instance, most studies have been performed in animals but they still need to be validated in human samples. Moreover, most studies considered only one or few growth factors, whereas microarray and/or proteomic approaches could permit contemporary analyses of large groups of growth factors. We did not include in this review study the trophic and plasticity effects of classic neurotransmitters/neuromodulators, but it is important to stress that these factors are also pivotal in determining and modulating the developmental and environmental changes of CB.

## Figures and Tables

**Table 1:** Nerve growth factor family. Expression of NGF, BDNF, and NT-3 in the carotid body.

Growth Factor	Localization	Species	Detection Methods	Reference
NGF	Type I cells Type II cells	Rat	Immunohistochemistry	Atanasova and Lazarov, 2014 [20]
BDNF	Carotid body	Rat	RT-PCR Southern blots	Hertzberg et al., 1994 [21]
	Carotid body Nerve fibers		In situ hybridization Immunohistochemistry	Brady et al., 1999 [22]
	Type I cells		Double immunofluorescence	Izal-Azcárate et al., 2008 [23]
	Carotid body		RT-PCR Western blot	Bairam et al., 2010 [29]
	Carotid body		qRT-PCR ELISA	Dmitrieff et al., 2011 [25]
	Carotid body		qRT-PCR Western blot ELISA	Chavez-Valdez et al. 2012 [28]
	Type I cells Type II cells		Immunohistochemistry	Atanasova and Lazarov, 2014 [20]
NT-3	Type I cells Type II cells	Rat	Immunohistochemistry	Atanasova and Lazarov, 2014 [20]

**Table 2:** Nerve growth factor family receptors. Expression of TrkA, TrkB, TrkC, and p75 in the carotid body.

Growth Factor	Receptor	Localization	Species	Detection Methods	Reference
NGF	TrkA p75	Type I cells Type II cells	Rat	Immunohistochemistry	Atanasova and Lazarov, 2014 [20]
BDNF	p75	Type I cells Type II cells	Rat	Double immunofluorescence	Izal-Azcárate et al., 2008 [23]
	TrkB	Carotid body		RT-PCR Western blot	Bairam et al., 2010 [29]
	TrkB	Carotid body		Western blot	Dmitrieff et al., 2011 [25]
	TrkB p75	Carotid body		qRT-PCR Western blot	Chavez-Valdez et al. 2012 [28]
	TrkB p75	Type I cells Type II cells		Immunohistochemistry	Atanasova and Lazarov, 2014 [20]
NT-3	TrkC p75	Type I cells Type II cells	Rat	Immunohistochemistry	Atanasova and Lazarov, 2014 [20]

**Table 3:** Glial cell line-derived neurotrophic factor family. Expression of GDNF and ARTN in the carotid body.

Growth Factor	Localization	Species	Detection Methods	Reference
GDNF	Carotid body	Rat	In situ hybridization	Nosrat et al., 1996 [32]
	Carotid body	Rat	ELISA	Lipton et al., 1999 [33]
	Carotid body	Mouse	Immunohistochemistry	Erickson et al., 2001 [37]
	Carotid body Type I cells	Rat	RT-PCR X-gal staining	Toledo-Aral et al., 2003 [34]
	Carotid body	Rat	RT-PCR	Leitner et al., 2005 [35]
	Type I cells	Mouse	X-gal staining	Villadiego et al., 2005 [36]
	Carotid body	Rat	Standard and in situ ELISA	
	Carotid body	Mouse	RT-PCR	Balbir et al., 2006 [38]
	Carotid body	Mouse	Microarray analysis	Balbir et al., 2007 [39]
	Type I cells	Rat	Double immunofluorescence	Izal-Azcárate et al., 2008 [23]
	Carotid body	Rat	qRT-PCR ELISA	Dmitrieff et al., 2011 [25]
	Carotid body cell aggregates	Rat	ELISA	Rodriguez-Pallares et al., 2012 [45]
	Carotid body	Human Rat	RT-PCR ELISA	Ortega-Sáenz et al., 2013 [42]
	Carotid body	Mouse	qRT-PCR X-gal staining	Muñoz-Manchado et al., 2013 [46]
	Type I cells	Rat	Immunohistochemistry	Atanasova and Lazarov, 2014 [20]
ARTN	Carotid body	Rat	RT-PCR	Leitner et al., 2005 [35]

**Table 4:** Glial cell line-derived neurotrophic factor family receptors. Expression of GFRα1-3 and RET in the carotid body.

Growth Factor	Receptor	Localization	Species	Detection Methods	Reference
GDNF	GFRα1 RET	Carotid body	Rat	RT-PCR	Toledo-Aral et al., 2003 [34]
	GFRα1 RET	Carotid body	Rat	RT-PCR Immunohistochemistry	Leitner et al., 2005 [35]
	RET	Type I cells	Rat	Double immunofluorescence	Izal-Azcárate et al., 2008 [23]
	RET	Carotid body	Rat	qRT-PCR	Dmitrieff et al., 2011 [25]
	GFRα1	Type I cells	Rat	Immunohistochemistry	Atanasova and Lazarov, 2014 [20]
NRTN	GFRα2	Carotid body	Rat	RT-PCR Immunohistochemistry	Leitner et al., 2005 [35]
ARTN	GFRα3	Carotid body	Rat	RT-PCR Immunohistochemistry	Leitner et al., 2005 [35]

**Table 5:** Ciliary neurotrophic factor family and insulin-like growth factor family. Expression of CNTF, IGF-I, and IGF-II in the carotid body.

Growth Factor	Localization	Species	Detection Methods	Reference
CNTF	Type I cells	Rat	Double immunofluorescence	Izal-Azcárate et al., 2008 [23]
IGF-I	Type I cells	Rat	Double immunofluorescence	Izal-Azcárate et al., 2008 [23]
IGF-II	Type I cells	Human	Immunohistochemistry	Suzuki et al., 1989 [53]

**Table 6:** Fibroblast growth factor, epidermal growth factor/transforming growth factor-α family, and transforming growth factor-β. Expression of bFGF, EGF, TGF-α, TGF-β, and BMP2 in the carotid body.

Growth Factor	Localization	Species	Detection Methods	Reference
bFGF	Type I cells	Rat	Immunofluorescence	Paciga and Nurse, 2001 [58]
	Type I cells Type II cells	Rat	Immunohistochemistry	Cao et al., 2003 [60]
	Type I cells	Human	qRT-PCR Immunohistochemistry	Douwes Dekker et al., 2007 [59]
	Type I cells Type II cells	Rat	Double immunofluorescence	Izal-Azcárate et al., 2008 [23]
EGF	Type I cells	Rat	Double immunofluorescence	Izal-Azcárate et al., 2008 [23] Belzunegui et al., 2008 [61]
TGF-α	Type I cells	Rat	Double immunofluorescence	Izal-Azcárate et al., 2008 [23]
TGF-β1	Carotid body	Rat	Immunohistochemistry	Milei et al., 2004 [62] Toblli et al., 2007 [63]
TGF-β2	Type I cells	Rat	Immunohistochemistry	Cao et al., 2003 [60]
BMP2	Carotid body	Mouse	qRT-PCR Microarray analysis	Balbir et al., 2007 [39]

**Table 7:** Ciliary neurotrophic factor family, fibroblast growth factor, epidermal growth factor family, and platelet-derived growth factor receptors. Expression of gp130, FGFR, EGFR, and PDGFR in the carotid body.

Growth Factor	Receptor	Localization	Species	Detection Methods	Reference
CNTF	gp130	Type I cells	Rat	RT-PCR Immunohistochemistry	Lam et al., 2008 [49]
bFGF	FGFR	Type I cells	Rat	Immunofluorescence	Paciga and Nurse, 2001 [58]
	FGFR1	Type I cells	Human	qRT-PCR Immunohistochemistry	Douwes Dekker et al., 2007 [59]
EGF	EGFR	Type I cells Type II cells	Rat	Double immunofluorescence	Izal-Azcárate et al., 2008 [23]
PDFG	PDGFR	Type I cells Type II cells	Rat	Double immunofluorescence	Izal-Azcárate et al., 2008 [23]

**Table 8:** Inflammatory cytokines. Expression of IL-1β, IL6, IL-4, IL-8, IL-10, and TNFα in the carotid body.

Growth Factor	Localization	Species	Detection Methods	Reference
IL-1α	Carotid body	Rat	qPCR RNA sequencing	Mkrtchian et al. 2020 [68]
IL-1β	Type I cells	Rat	RT-PCR Double immunofluorescence	Lam et al., 2008 [49]
	Type I cells	Rat	Amplified RNA/qPCR technology	Liu et al., 2009 [69]
	Carotid body	Rat	Immunohistochemistry	Del Rio et al., 2011 [71]
	Type I cells	Rat	RT-PCR Double immunofluorescence	Lam et al., 2012 [66]
	Carotid body	Rat	qRT-PCR	Liu et al., 2012 [67]
	Carotid body	Rat	Immunohistochemistry	Del Rio et al., 2012 [72]
	Type I cells	Rat	Immunohistochemistry	Del Rio et al., 2012 [73]
	Carotid body	Human	ELISA	Kåhlin et al., 2014 [76]
IL-6	Carotid body lysates	Rabbit	ELISA	Feng et al., 2008 [75]
	Type I cells	Rat	RT-PCR Double immunofluorescence	Lam et al., 2008 [49]
	Type I cells Type II cells	Rat	Amplified RNA/qPCR technology In situ hybridization	Liu et al., 2009 [69]
	Carotid body	Rat	Immunohistochemistry	Del Rio et al., 2011 [71]
	Type I cells	Rat	RT-PCR Double immunofluorescence	Lam et al., 2012 [66]
	Carotid body	Rat	qRT-PCR	Liu et al., 2012 [67]
	Carotid body	Human	ELISA	Kåhlin et al., 2014 [76]
IL-4, IL-8, IL-10	Carotid body	Human	ELISA	Kåhlin et al., 2014 [76]
TNFα	Type I cells	Rat	RT-PCR Double immunofluorescence	Lam et al., 2008 [49]
	Type I cells Endothelial cells	Cat	Immunohistochemistry	Fernandez et al., 2008 [70]
	Type I cells		Amplified RNA/qPCR technology	Liu et al., 2009 [69]
	Type I cells	Rat	RT-PCR Double immunofluorescence Western blot	Fernandez et al., 2011 [65]
	Carotid body	Rat	Immunohistochemistry	Del Rio et al., 2011 [71]
	Type I cells	Rat	RT-PCR Double immunofluorescence	Lam et al., 2012 [66]
	Carotid body	Rat	qRT-PCR	Liu et al., 2012 [67]
	Carotid body	Rat	Immunohistochemistry	Del Rio et al., 2012 [72]
	Type I cells	Rat	Immunohistochemistry	Del Rio et al., 2012 [73]
	Carotid body	Rat	ELISA	Mkrtchian et al. 2020 [68]

**Table 9:** IL-1β, IL-6, and TNFα receptors. Expression of in the carotid body.

Growth Factor	Receptor	Localization	Species	Detection Methods	Reference
IL-1β	IL-1R1	Type I cells	Rat	Double immunofluorescence Western blot	Zhang et al., 2007 [74]
		Type I cells	Rat	RT-PCR Double immunofluorescence	Lam et al., 2008 [49]
		Type I cells	Rat	RT-PCR Double immunofluorescence	Lam et al., 2012 [66]
		Type I cells	Human	Double immunofluorescence	Kåhlin et al., 2014 [76]
IL-6	IL-6Rα	Type I cells	Rat	RT-PCR Double immunofluorescence Western blot	Wang et al., 2006 [50]
	gp130	Type I cells	Rat	RT-PCR Double immunofluorescence Western blot	Wang et al., 2006 [50]
		Type I cells	Rat	RT-PCR Double immunofluorescence	Lam et al., 2008 [49]
		Type I cells	Rat	RT-PCR Double immunofluorescence	Lam et al., 2012 [66]
		Type I cells	Human	Double immunofluorescence	Kåhlin et al., 2014 [76]
TNFα	TNFR1	Type I cells	Rat	RT-PCR Double immunofluorescence	Lam et al., 2008 [49]
	TNFR1	Type I cells	Cat	RT-PCR Immunohistochemistry Western blot	Fernandez et al., 2008 [70]
	TNFR2	Endothelial cells	Cat	RT-PCR Immunohistochemistry	
	TNFR1	Type I cells	Rat	RT-PCR Double immunofluorescence Western blot	Fernandez et al., 2011 [65]
	TNFR2	Surrounding glomus cell clusters			
	TNFR1	Type I cells	Rat	RT-PCR Double immunofluorescence	Lam et al., 2012 [66]

**Table 10:** Vascular growth factor. Expression of VEGF in the carotid body.

Growth Factor	Localization	Species	Detection Methods	Reference
VEGF	Type I cells	Rat	Immunohistochemistry	Tipoe and Fung, 2003 [83] Lam et al., 2008 [87]
	Type I cells	Rat	Immunocytochemistry	Chen et al., 2003 [84]
	Carotid body	Rat	Immunohistochemistry	Di Giulio et al., 2003 [85] Di Giulio et al., 2005 [86] Di Giulio et al., 2009 [88]
	Carotid body lysates	Rabbit	ELISA	Feng et al., 2008 [75]
	Type I cells	Rat	Double immunofluorescence	Belzunegui et al., 2008 [61]
	Type I cells Blood vessels	Rat	Immunohistochemistry	Del Rio et al., 2011 [89] Felix et al., 2012 [90]
	Carotid body	Human	Immunohistochemistry	Zara et al., 2013 [92] Zara et al., 2013 [93]
	Carotid body	Rat	qRT-PCR	Salman et al., 2017 [91]

**Table 11:** Endothelins. Expression of ET-1 in the carotid body.

Growth Factor	Localization	Species	Detection Methods	Reference
ET-1	Type I cells	Rat	Immunocytochemistry	He et al., 1996 [96]
	Endothelial cells	Rat	Immunohistochemistry Immunoelectron microscopy	Ozaka et al., 1997 [94]
	Type I cells	Rat	qRT-PCR Immunocytochemistry	Chen et al., 2002 [97]
	Type I cells	Rat	Immunohistochemistry	Lam et al., 2008 [87]
	Carotid body lysates	Rabbit	ELISA	Feng et al., 2008 [75]
	Type I cells Endothelium of blood vessels	Cat	Immunohistochemistry	Rey et al., 2006 [99]
	Type I cells Perilobular areas	Cat	Immunohistochemistry	Rey et al., 2006 [100] Rey et al., 2008 [102]
	Type I cells Blood vessels	Cat	Double immunofluorescence	Rey et al., 2007 [101]
	Type I cells	Rat	RT-PCR Double immunofluorescence EIA	Pawar et al., 2009 [103]
	Blood vessels Carotid body parenchyma	Rat	Immunohistochemistry	Di Giulio et al., 2009 [107]
	Type I cells Perilobular areas	Rat	Immunohistochemistry	Del Rio et al., 2011 [71]
	Type I cells Intralobular immune cells	Rat	Double immunofluorescence	Liu et al., 2012 [67]
	Carotid body	Rat	qRT-PCR	Liu et al., 2013 [104]
	Carotid body Type I cells Blood vessels	Rat	qRT-PCR Double immunofluorescence EIA	Peng et al., 2013 [105]
	Carotid body	Rat	cDNA expression array	Mosqueira and Iturriaga, 2019 [106]

**Table 12:** Vascular growth factor and endothelin receptors. Expression of VEGFR1, VEGFR2, Flk-1, ET_A_-R and ET_B_-R in the carotid body.

Growth Factor	Receptor	Localization	Species	Detection Methods	Reference
VEGF	VEGFR1 VEGFR2	Carotid body	Rat	Immunohistochemistry	Tipoe and Fung, 2003 [83]
	Flk-1	Type I cells	Rat	Immunocytochemistry	Chen et al., 2003 [84]
ET-1	ET_A_-R	Type I cells	Rat	qRT-PCR Immunocytochemistry	Chen et al., 2002 [97]
	ET_A_-R ET_B_-R	Type I cells Endothelium of blood vessels	Cat	Immunohistochemistry Western blot	Rey et al., 2007 [101] Rey et al., 2008 [102]
	ET_A_-R ET_B_-R	Carotid body	Rat	RT-PCR	Pawar et al., 2009 [103]
	ET_A_-R ET_B_-R	Carotid body	Rat	qRT-PCR	Peng et al., 2013 [105]
	ET_A_-R ET_B_-R	Carotid body	Rat	Western blot	Mosqueira and Iturriaga, 2019 [106]

## Abbreviations

6-OHDA	6-hydroxydopamine
ARTN	artemin
BDNF	brain-derived neurotrophic factor
BMP	morphogenetic protein
CA	catecholamine
CB	carotid body
CH	chronic hypoxia
CIH	chronic intermittent hypoxia
CNTF	ciliary neurotrophic factor
DA	dopaminergic
DAMPs	damage-associated molecular patterns
ECE	endothelin-converting enzyme
EGF	epidermal growth factor
EIA	enzyme immunoassay
ERK	extracellular signal-regulated kinase
ET	endothelin
FGF	fibroblast growth factor
GDNF	glial cell line-derived neurotrophic factor
GFLs	GDNF family ligands
GFR	glycosyl-phosphatidylinositol-anchored coreceptor
gp130	glycoprotein 130
GPCRs	G-protein coupled receptors
HIF-1α	hypoxia inducible factor-1α
IGF	insulin-like growth factor
IH/ROX	intermittent hypoxia/reoxygenation
IHH	intermittent hypobaric hypoxia
IL	Interleukin
JAK/STAT	Janus kinase/signal transduction and activator of transcription
LIF	leukemia inhibitory factor
L-NAME	hydrochloride NG-nitro-methyl ester-L-arginine
LPA	lysophosphatidic acid
LPS	lipopolysaccharide
MCP-1	monocyte chemoattractant protein-1
MPTP	1-methyl-4-phenyl-1,2,3,6,-tetahydropyridine
NCAM	neural cell adhesion molecule
NCT	neonatal caffeine treatment
NGF	nerve growth factor
NMDA	N-methyl-D-aspartate
NOS	nitric oxide synthase
NRTN	neurturin
NSCs	neural stem cells
NT	neurotrophin
PD	Parkinson’s disease
PDGF	platelet-derived growth factor
PLA	proximity ligation assay
PSPN	persephin
RET	Ret tyrosine kinase receptor
RRI	receptor–receptor interactions
RTKs	receptor tyrosine kinases
SCG	superior cervical ganglion
SHR	spontaneously hypertensive rats
SVZ/OB	subventricular zone/olfactory bulb
TGF	transforming growth factor
TH	tyrosine hydroxylase
TNFα	tumor necrosis factor α
Trk	tyrosine receptor kinase
VEGF	vascular endothelial growth factor
VM	ventral mesencephalon
